# 1-Methyl-4-[(1*E*)-2-nitro­prop-1-en-1-yl]benzene

**DOI:** 10.1107/S1600536812025913

**Published:** 2012-06-13

**Authors:** Zhao-Bo Li, Li-Li Shen, Jian-An Zheng

**Affiliations:** a Hangzhou Minsheng Pharmaceutical Group Co. Ltd, Hangzhou 310000, People’s Republic of China; b State Key Laboratory Breeding Base of Green Chemistry–Synthesis Technology, Zhejiang University of Technology, Hangzhou 310014, People’s Republic of China; c Jiaxing Zhonghua Chemical Industry Co. Ltd, Daqiao Town Nanhu District, Jiaxing 314006, People’s Republic of China

## Abstract

The title compound, C_10_H_11_NO_2_, adopts an *E* conformation about the C=C bond. The C=C—C=C torsion angle is 32.5 (3)°. The crystal structure features weak inter­molecular C—H⋯O inter­actions.

## Related literature

For background to the chemistry of nitro­alkenes, see: Ballini & Petrini (2004 ▶); Berner *et al.* (2002 ▶); Ono (2001 ▶). For a related structure, see: Yang *et al.* (2010 ▶).
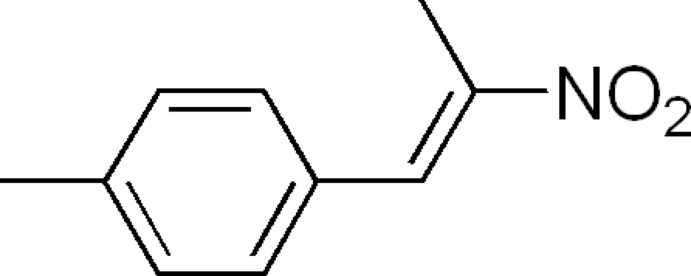


## Experimental

### 

#### Crystal data

C_10_H_11_NO_2_
*M* *_r_* = 177.20
Orthorhombic, 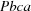

*a* = 11.0610 (5) Å
*b* = 7.5840 (4) Å
*c* = 22.6420 (11) Å
*V* = 1899.36 (16) Å^3^
*Z* = 8
Mo *K*α radiation
μ = 0.09 mm^−1^
*T* = 296 K
0.58 × 0.43 × 0.36 mm

#### Data collection

Rigaku R-AXIS-RAPID/ZJUG diffractometer
Absorption correction: multi-scan (*ABSCOR*; Higashi, 1995 ▶) *T*_min_ = 0.941, *T*_max_ = 0.969
16972 measured reflections
2162 independent reflections
1325 reflections with *I* > 2σ(*I*)
*R*_int_ = 0.033

#### Refinement

*R*[*F* ^2^ > 2σ(*F* ^2^)] = 0.045
*wR*(*F* ^2^) = 0.134
*S* = 1.00
2162 reflections
121 parameters
H-atom parameters constrained
Δρ_max_ = 0.15 e Å^−3^
Δρ_min_ = −0.17 e Å^−3^

### 

Data collection: *PROCESS-AUTO* (Rigaku, 2006 ▶); cell refinement: *PROCESS-AUTO*; data reduction: *CrystalClear* (Rigaku, 2007 ▶); program(s) used to solve structure: *SHELXS97* (Sheldrick, 2008 ▶); program(s) used to refine structure: *SHELXL97* (Sheldrick, 2008 ▶); molecular graphics: *ORTEP-3 for Windows* (Farrugia, 1997 ▶); software used to prepare material for publication: *WinGX* (Farrugia, 1999 ▶).

## Supplementary Material

Crystal structure: contains datablock(s) I. DOI: 10.1107/S1600536812025913/zq2167sup1.cif

Structure factors: contains datablock(s) I. DOI: 10.1107/S1600536812025913/zq2167Isup2.hkl

Supplementary material file. DOI: 10.1107/S1600536812025913/zq2167Isup3.cml

Additional supplementary materials:  crystallographic information; 3D view; checkCIF report

## Figures and Tables

**Table 1:** Hydrogen-bond geometry (Å, °)

*D*—H⋯*A*	*D*—H	H⋯*A*	*D*⋯*A*	*D*—H⋯*A*
C8—H8⋯O1^i^	0.93	2.55	3.369 (2)	147
C2—H2⋯O2^ii^	0.93	2.66	3.551 (3)	162

Symmetry codes: (i) 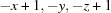; (ii) 
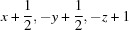
.

**Fig. 1.**
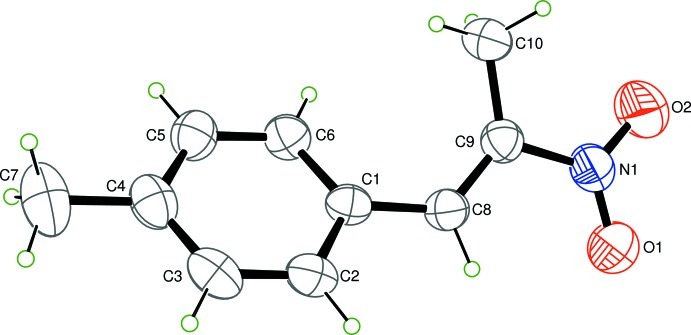
The asymmetric unit of the title compound with the atomic labeling scheme; displacement ellipsoids are drawn at the 50% probability level.

**Fig. 2.**
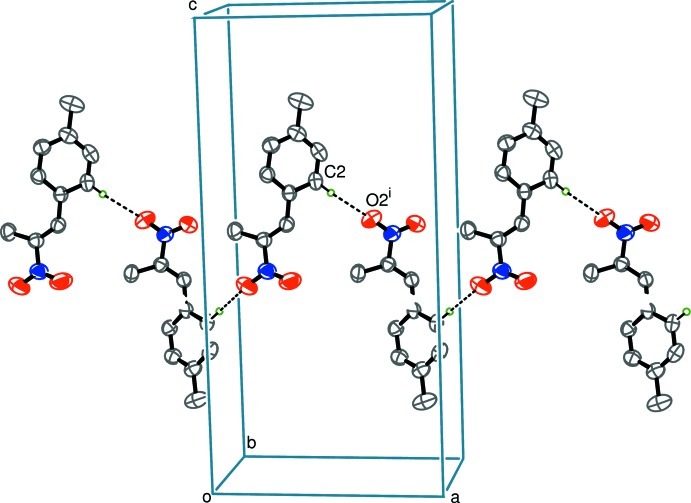
View of the intermolecular interactions illustrated as dashed lines.

## supplementary crystallographic information

### Comment

Nitroalkenes are important organic intermediates, since they can be converted to synthetically useful N- and O-containing organic molecules, such as amines, aldehydes, carboxylic acids, or denitrated compounds (Ono, 2001; Berner *et al.*, 2002; Ballini & Petrini, 2004). As a contribution in this field, we have synthesized a series of nitroalkenes by employing benzaldehydes and nitroethane (Yang *et al.*, 2010). We report here the crystal structure of the title compound (Fig. 1).

The molecule adopts an *E* configuration with respect to the C8=C9 double bond. The torsion angle C9—C8—C1—C6 is 32.5 (3)°. In the crystal structure, the molecules interact through weak intermolecular C8—H8···O1^i^ and C2—H2···O2^ii^ hydrogen bonds (symmetry codes: *i* = -*x*+1, -*y*, -*z*+1; *ii* = *x*+1/2, -*y*+1/2, -*z*+1; Fig. 2 and Table 1).

### Experimental

To a solution of *p*-tolualdehyde (50 mmol) in AcOH (25 mL), nitroethane (75 mmol) was added, followed by butylamine (100 mmol, 7.4 mL). The mixture was sonicated at 60 °C, until GC showed full conversion of the aldehyde. The mixture was poured into ice water, the precipitate was filtered off, washed with water and recrystallized from EtOH/EtOAc to give the final product. Single crystals were obtained by slow evaporation of a *n*-hexane/EtOAc (10:1, *v*/*v*) solution.

### Refinement

All H atoms were placed in calculated positions and refined using a riding model, with C—H = 0.93 Å and *U*_iso_(H) = 1.2*U*_eq_(C) for aromatic H atoms, and with C—H = 0.96 Å and *U*_iso_(H) = 1.5*U*_eq_(C) for methyl H atoms.

### Crystal data

**Table:**

C_10_H_11_NO_2_	*F*(000) = 752
*M**_r_* = 177.20	*D*_x_ = 1.239 Mg m^−^^3^
Orthorhombic, *P**b**c**a*	Mo *K*α radiation, λ = 0.71073 Å
Hall symbol: -P 2ac 2ab	Cell parameters from 9467 reflections
*a* = 11.0610 (5) Å	θ = 3.2–27.4°
*b* = 7.5840 (4) Å	µ = 0.09 mm^−^^1^
*c* = 22.6420 (11) Å	*T* = 296 K
*V* = 1899.36 (16) Å^3^	Chunk, yellow
*Z* = 8	0.58 × 0.43 × 0.36 mm

### Data collection

**Table:**

Rigaku R-AXIS-RAPID/ZJUG diffractometer	2162 independent reflections
Radiation source: rotating anode	1325 reflections with *I* > 2σ(*I*)
Graphite monochromator	*R*_int_ = 0.033
Detector resolution: 10.00 pixels mm^-1^	θ_max_ = 27.4°, θ_min_ = 3.4°
ω scans	*h* = −14→14
Absorption correction: multi-scan (*ABSCOR*; Higashi, 1995)	*k* = −9→9
*T*_min_ = 0.941, *T*_max_ = 0.969	*l* = −29→29
16972 measured reflections	

### Refinement

**Table:**

Refinement on *F*^2^	Secondary atom site location: difference Fourier map
Least-squares matrix: full	Hydrogen site location: inferred from neighbouring sites
*R*[*F*^2^ > 2σ(*F*^2^)] = 0.045	H-atom parameters constrained
*wR*(*F*^2^) = 0.134	*w* = 1/[σ^2^(*F*_o_^2^) + (0.0552*P*)^2^ + 0.4885*P*] where *P* = (*F*_o_^2^ + 2*F*_c_^2^)/3
*S* = 1.00	(Δ/σ)_max_ = 0.001
2162 reflections	Δρ_max_ = 0.15 e Å^−^^3^
121 parameters	Δρ_min_ = −0.17 e Å^−^^3^
0 restraints	Extinction correction: *SHELXL*, Fc^*^=kFc[1+0.001xFc^2^λ^3^/sin(2θ)]^-1/4^
Primary atom site location: structure-invariant direct methods	Extinction coefficient: 0.0051 (12)

### Special details

**Table:**

Geometry. All e.s.d.'s (except the e.s.d. in the dihedral angle between two l.s. planes) are estimated using the full covariance matrix. The cell e.s.d.'s are taken into account individually in the estimation of e.s.d.'s in distances, angles and torsion angles; correlations between e.s.d.'s in cell parameters are only used when they are defined by crystal symmetry. An approximate (isotropic) treatment of cell e.s.d.'s is used for estimating e.s.d.'s involving l.s. planes.
Refinement. Refinement of *F*^2^ against ALL reflections. The weighted *R*-factor *wR* and goodness of fit *S* are based on *F*^2^, conventional *R*-factors *R* are based on *F*, with *F* set to zero for negative *F*^2^. The threshold expression of *F*^2^ > σ(*F*^2^) is used only for calculating *R*-factors(gt) *etc*. and is not relevant to the choice of reflections for refinement. *R*-factors based on *F*^2^ are statistically about twice as large as those based on *F*, and *R*- factors based on ALL data will be even larger.

### Fractional atomic coordinates and isotropic or equivalent isotropic displacement parameters (Å^2^)

**Table:**

	*x*	*y*	*z*	*U*_iso_*/*U*_eq_	
C1	0.35993 (13)	0.1487 (2)	0.63005 (7)	0.0487 (4)	
C2	0.46387 (14)	0.2326 (2)	0.65095 (8)	0.0577 (4)	
H2	0.5248	0.2634	0.6246	0.069*	
C3	0.47750 (16)	0.2703 (2)	0.71015 (8)	0.0643 (5)	
H3	0.5464	0.3300	0.7227	0.077*	
C4	0.39139 (17)	0.2218 (2)	0.75151 (8)	0.0614 (5)	
C5	0.29031 (16)	0.1324 (2)	0.73072 (8)	0.0615 (5)	
H5	0.2318	0.0957	0.7575	0.074*	
C6	0.27416 (14)	0.0963 (2)	0.67157 (7)	0.0547 (4)	
H6	0.2053	0.0363	0.6592	0.066*	
C7	0.4073 (2)	0.2610 (3)	0.81617 (9)	0.0938 (7)	
H7A	0.4047	0.1529	0.8382	0.141*	
H7B	0.3435	0.3374	0.8292	0.141*	
H7C	0.4839	0.3177	0.8223	0.141*	
C8	0.34896 (14)	0.1144 (2)	0.56645 (7)	0.0531 (4)	
H8	0.4211	0.0963	0.5462	0.064*	
C9	0.24878 (15)	0.1058 (2)	0.53415 (7)	0.0524 (4)	
C10	0.12094 (14)	0.1404 (3)	0.55103 (8)	0.0676 (5)	
H10A	0.0791	0.0304	0.5557	0.101*	
H10B	0.0824	0.2087	0.5207	0.101*	
H10C	0.1189	0.2043	0.5876	0.101*	
N1	0.26510 (15)	0.0587 (2)	0.47133 (7)	0.0689 (4)	
O1	0.36592 (15)	0.0320 (3)	0.45180 (6)	0.1156 (7)	
O2	0.17592 (15)	0.0466 (3)	0.44070 (7)	0.1107 (6)	

### Atomic displacement parameters (Å^2^)

**Table:**

	*U*^11^	*U*^22^	*U*^33^	*U*^12^	*U*^13^	*U*^23^
C1	0.0436 (8)	0.0448 (8)	0.0577 (9)	0.0026 (6)	−0.0012 (7)	0.0014 (7)
C2	0.0443 (8)	0.0582 (10)	0.0705 (11)	−0.0013 (7)	−0.0040 (8)	0.0051 (8)
C3	0.0588 (10)	0.0554 (10)	0.0788 (12)	−0.0025 (8)	−0.0221 (9)	−0.0002 (8)
C4	0.0758 (11)	0.0482 (9)	0.0602 (10)	0.0081 (8)	−0.0150 (9)	−0.0001 (8)
C5	0.0675 (11)	0.0592 (10)	0.0576 (10)	−0.0005 (8)	0.0030 (8)	0.0056 (8)
C6	0.0527 (9)	0.0527 (9)	0.0587 (9)	−0.0081 (7)	−0.0020 (7)	0.0009 (7)
C7	0.134 (2)	0.0821 (14)	0.0653 (12)	0.0051 (14)	−0.0291 (13)	−0.0057 (10)
C8	0.0474 (8)	0.0563 (9)	0.0555 (9)	0.0029 (7)	0.0031 (7)	0.0019 (7)
C9	0.0535 (8)	0.0525 (9)	0.0512 (8)	0.0023 (7)	−0.0009 (7)	0.0009 (7)
C10	0.0500 (9)	0.0809 (12)	0.0718 (11)	0.0055 (9)	−0.0033 (8)	0.0057 (9)
N1	0.0687 (10)	0.0811 (11)	0.0570 (9)	0.0123 (8)	−0.0067 (8)	−0.0012 (8)
O1	0.0869 (11)	0.197 (2)	0.0625 (9)	0.0435 (12)	0.0054 (8)	−0.0159 (10)
O2	0.0889 (11)	0.1695 (18)	0.0737 (10)	0.0049 (11)	−0.0255 (8)	−0.0238 (10)

### Geometric parameters (Å, º)

**Table:**

C1—C2	1.396 (2)	C7—H7A	0.9600
C1—C6	1.394 (2)	C7—H7B	0.9600
C1—C8	1.468 (2)	C7—H7C	0.9600
C2—C3	1.379 (2)	C8—C9	1.329 (2)
C2—H2	0.9300	C8—H8	0.9300
C3—C4	1.386 (3)	C9—N1	1.478 (2)
C3—H3	0.9300	C9—C10	1.488 (2)
C4—C5	1.390 (2)	C10—H10A	0.9600
C4—C7	1.504 (3)	C10—H10B	0.9600
C5—C6	1.378 (2)	C10—H10C	0.9600
C5—H5	0.9300	N1—O2	1.2094 (19)
C6—H6	0.9300	N1—O1	1.217 (2)
			
C2—C1—C6	117.50 (15)	H7A—C7—H7B	109.5
C2—C1—C8	118.77 (14)	C4—C7—H7C	109.5
C6—C1—C8	123.69 (14)	H7A—C7—H7C	109.5
C3—C2—C1	120.94 (16)	H7B—C7—H7C	109.5
C3—C2—H2	119.5	C9—C8—C1	128.09 (14)
C1—C2—H2	119.5	C9—C8—H8	116.0
C2—C3—C4	121.78 (16)	C1—C8—H8	116.0
C2—C3—H3	119.1	C8—C9—N1	116.07 (14)
C4—C3—H3	119.1	C8—C9—C10	129.97 (15)
C3—C4—C5	116.99 (16)	N1—C9—C10	113.95 (14)
C3—C4—C7	121.66 (18)	C9—C10—H10A	109.5
C5—C4—C7	121.35 (19)	C9—C10—H10B	109.5
C6—C5—C4	122.02 (16)	H10A—C10—H10B	109.5
C6—C5—H5	119.0	C9—C10—H10C	109.5
C4—C5—H5	119.0	H10A—C10—H10C	109.5
C5—C6—C1	120.70 (15)	H10B—C10—H10C	109.5
C5—C6—H6	119.7	O2—N1—O1	121.78 (17)
C1—C6—H6	119.7	O2—N1—C9	118.09 (16)
C4—C7—H7A	109.5	O1—N1—C9	120.13 (15)
C4—C7—H7B	109.5		
			
C6—C1—C2—C3	−3.5 (2)	C8—C1—C6—C5	179.77 (15)
C8—C1—C2—C3	178.93 (15)	C2—C1—C8—C9	−150.12 (16)
C1—C2—C3—C4	2.3 (3)	C6—C1—C8—C9	32.5 (3)
C2—C3—C4—C5	0.2 (2)	C1—C8—C9—N1	−176.58 (15)
C2—C3—C4—C7	179.15 (17)	C1—C8—C9—C10	4.9 (3)
C3—C4—C5—C6	−1.4 (2)	C8—C9—N1—O2	178.77 (17)
C7—C4—C5—C6	179.65 (17)	C10—C9—N1—O2	−2.5 (2)
C4—C5—C6—C1	0.1 (3)	C8—C9—N1—O1	−0.8 (3)
C2—C1—C6—C5	2.3 (2)	C10—C9—N1—O1	177.99 (18)

### Hydrogen-bond geometry (Å, º)

**Table:**

*D*—H···*A*	*D*—H	H···*A*	*D*···*A*	*D*—H···*A*
C8—H8···O1	0.93	2.28	2.677 (2)	105
C8—H8···O1^i^	0.93	2.55	3.369 (2)	147
C2—H2···O2^ii^	0.93	2.66	3.551 (3)	162

Symmetry codes: (i) −*x*+1, −*y*, −*z*+1; (ii) *x*+1/2, −*y*+1/2, −*z*+1.
